# Early respiratory deterioration predicts poor outcome after aneurysmal subarachnoid hemorrhage

**DOI:** 10.1186/cc12276

**Published:** 2013-03-19

**Authors:** E Van Lummel, L Vergouw, M Van der Jagt

**Affiliations:** 1 Erasmus MC, Rotterdam, the Netherlands

## Introduction

Pulmonary complications are frequently occurring medical complications after aneurysmal subarachnoid hemorrhage (aSAH) [1]. Early respiratory deterioration (ERD) may be associated with delayed cerebral ischemia (DCI) or outcome and would then be a potential target for therapeutic interventions. We investigated whether respiratory deterioration within the first 72 hours after admission predicted DCI or poor outcome.

## Methods

We conducted a retrospective study in 137 consecutively admitted patients with aSAH, admitted between October 2007 and October 2011 to the ICU of a university hospital. ERD was defined as increased need for ventilatory support the second or third day after admission (Table 1). Elective intubation for a surgical procedure was not included as ERD. Inclusion criteria were availability of detailed information on respiratory status and level of support, admission within 48 hours after hemorrhage and age ≥18 years. Multivariable survival analysis was used to investigate associations of DCI, death and Glasgow Outcome Scale (GOS) with ERD adjusted for condition on admission, Hijdra score, treatment of ruptured aneurysm and pulmonary comorbidity. GOS was assessed at 3 to 6 months after the bleed. DCI was defined as described recently [2].

## Results

Mean age of the patients was 55.9 (± 12.7) and 63.5% was female. A total 46.7% of the patients developed DCI. Mortality was 25.6%. Forty percent of the patients were classified as having ERD. ERD was not associated with DCI (adjusted HR = 1.48; 95% CI = 0.88 to 2.49; *P *= 0.14). ERD showed a trend towards an association with mortality (adjusted HR = 2.22; 95% CI = 0.96 to 5.14; *P *= 0.06; additionally adjusted for age, and rebleed). A clear association was found between absence of ERD and functional outcome with ordinal logistic regression analysis

(0.98 point increase in GOS score at 3 to 6 months; 95% CI = 0.14 to 1.82; *P *= 0.02; additionally adjusted for age and rebleed).

## Conclusion

ERD within 72 hours after admission is associated with increased risk of poor functional outcome after aSAH, but not DCI. Further investigations are required to assess whether prevention of ERD may improve outcome.

**Table 1:** Categories of respiratory support

Category	Level of respiratory support (compared with first admission day)
1	Up to 5 l O_2_
2	>5 l O_2 _by nasal canulla or Venturi mask
3	Nonrebreathing mask or non-invasive positive-pressure ventilation
4	Invasive mechanical ventilation
When mechanically terminated	Decreased PaO_2_/FiO_2 _ratio
